# The utility of Xpert MTB/RIF performed on bronchial washings obtained in patients with suspected pulmonary tuberculosis in a high prevalence setting

**DOI:** 10.1186/s12890-015-0086-z

**Published:** 2015-09-16

**Authors:** Dewald A. Barnard, Elvis M. Irusen, Johannes W. Bruwer, Danté Plekker, Andrew C. Whitelaw, Jacobus D. Deetlefs, Coenraad F. N. Koegelenberg

**Affiliations:** Divisions of Pulmonology, Department of Medicine, Stellenbosch University, PO Box 19063, 7505 Cape Town, South Africa; Tygerberg Academic Hospital, Cape Town, South Africa; Kuils River Hospital, Cape Town, South Africa; Division of Medical Microbiology and Immunology, Department of Pathology, Stellenbosch University, Cape Town, South Africa; National Health Laboratory Services, Cape Town, South Africa; Ampath Laboratories, Cape Town, South Africa

## Abstract

**Background:**

Xpert MTB/RIF has been shown to have a superior sensitivity to microscopy for acid fast bacilli (AFB) in sputum and has been recommended as a standard first line investigation for pulmonary tuberculosis (PTB). Bronchoscopy is a valuable tool in diagnosing PTB in sputum negative patients. There is limited data on the utility of Xpert MTB/RIF performed on bronchial lavage specimens. Our aim was to evaluate the diagnostic efficiency of Xpert MTB/RIF performed on bronchial washings in sputum scarce/negative patients with suspected PTB.

**Methods:**

All patients with a clinical and radiological suspicion of PTB who underwent bronchoscopy between January 2013 and April 2014 were included. The diagnostic efficiencies of Xpert MTB/RIF and microscopy for AFB were compared to culture for *Mycobacterium tuberculosis*.

**Results:**

Thirty nine of 112 patients were diagnosed with culture-positive PTB. Xpert MTB/RIF was positive in 36/39 with a sensitivity of 92.3 % (95 % CI 78–98 %) for PTB, which was superior to that of smear microscopy (41 %; 95 % CI 26.0–57.8 %, *p* = 0.005). The specificities of Xpert MTB/RIF and smear microscopy were 87.7 % (95 % CI 77.4–93.9 %) and 98.6 % (95 % CI 91.6 %–99.9 %) respectively. Xpert MTB/RIF had a positive predictive value of 80 % (95 % CI; 65–89.9 %) and negative predictive value of 95.5 % (95 % CI 86.6–98.8 %). 3/9 patients with Xpert MTB/RIF positive culture negative results were treated for PTB based on clinical and radiological findings.

**Conclusion:**

Xpert MTB/RIF has a higher sensitivity than smear microscopy and similar specificity for the immediate confirmation of PTB in specimens obtained by bronchial washing, and should be utilised in patients with a high suspicion of pulmonary tuberculosis.

## Background

Early diagnosis and treatment of pulmonary tuberculosis is essential in reducing the spread, morbidity, mortality and the escalating costs associated with advanced disease [1]. Microbiological confirmation of *Mycobacterium tuberculosis* disease can be challenging in patients with a low bacterial load or in those who do not expectorate. Bronchoscopy with washings, with or without biopsy, can aid diagnosis by providing adequate samples for testing [2, 3].

Most of the evidence confirming the utility of the newly WHO endorsed Xpert MTB/RIF has been based on studies performed on sputum samples [4, 5]. The reported sensitivity of 90.4 % is significantly higher than that of smear microscopy, which is around 28 % [4, 6]. Recent studies have also suggested that Xpert MTB/RIF’s sensitivity is not affected by HIV status [4, 7]. There is limited data validating the routine use of Xpert MTB/RIF on bronchial lavages, as the majority of cohorts contained low numbers of patients from whom bronchial lavages were obtained [4, 7–9].

The aim of this study was to evaluate the diagnostic efficiency of Xpert MTB/RIF performed on bronchial washing fluid obtained bronchoscopically from patients with a clinical and radiological suspicion of pulmonary tuberculosis.

## Methods

### Study population

We screened all smear negative/sputum scarce patients who underwent bronchoscopy as part of their work up for suspected pulmonary tuberculosis at Tygerberg Academic Hospital and Kuils River Hospital during the period from January 2013 to April 2014, and retrospectively included all patients with complete sets of medical, radiological and laboratory data. Tygerberg Academic Hospital is a tertiary hospital in Cape Town, South Africa. It is one of two referral centres and it renders a tertiary service to a population of approximately 3.6 million people. Kuilsrivier Hospital is a secondary hospital in Cape Town, offering private health care to patients. For the purposes of our study we considered a clinical suspicion of pulmonary tuberculosis if any two of the following were present: known HIV infection, persistent cough lasting > 3 weeks, haemoptysis, weight loss > 4 kg, intermittent fever > 3 weeks or drenching night sweats > 2 weeks. In addition, at least one of the following radiological criteria had to be present for inclusion: cavitation, diffuse infiltrates, hilar or mediastinal adenopathy. The study was approved by the Health Research Ethics Committee of Stellenbosch University (S13/04/063). A waiver of consent was approved due to the retrospective nature of the study.

### Bronchoscopy procedure

Flexible bronchoscopies were performed in dedicated suites under conscious sedation. Washings were obtained by instilling 10 ml of 0.9 % saline into a segment of an affected lobe and aspirating available fluid (repeated three times). Transbronchial biopsies and transbronchial needle aspiration of lymph nodes were performed at the discretion of the endoscopist.

### Microbiology

Samples were processed according to standard laboratory protocols by decontamination with N-acetyl-L-cysteine/NaOH, centrifugation and fluorescent microscopy for acid fast bacilli [10]. Decontaminated samples were inoculated in the BACTEC MGIT culture system, (Becton Dickinson, Sparks, Maryland, USA) and incubated for 6 weeks [11]. Positive cultures were identified as *M. tuberculosis* and tested for susceptibility to rifampicin and isoniazid using the MTBDRplus line probe assay (Hain LifeSciences, Nehren, Germany).

All Xpert MTB/RIF samples were processed according to the manufacturer’s specifications [12]. Bronchial washing fluid was incubated, without prior decontamination, with sample reagent in a cartridge and loaded within 30 min of preparation. A test was positive if *Mycobacterium tuberculosis* was identified within 38 cycles [13]. Xpert MTB/RIF generated results (cycle threshold values) were categorised as either high positive (< 16), medium (16–21), low (22–28) or very low positive (> 28) [12].

### Diagnosis of pulmonary tuberculosis

We use a positive culture of *Mycobacterium tuberculosis* as the gold standard for the diagnosis of pulmonary tuberculosis in our primary analysis. In a secondary analysis, we also included cases with histological or cytological features of caseating granuloma containing acid fast bacilli, or cases where at least three clinical and one radiological criterion were present in a patient who subsequently showed documented clinical and radiological improvement after six months of anti-tuberculosis treatment (despite negative microbiology).

### Statistical analysis

Descriptive statistics were performed and a composite reference standard used. Analyses were conducted of data collected per patient. The sensitivity, specificity, positive predictive value (PPV) and negative predictive value (NPV) of Xpert MTB/RIF were calculated using standard methods and Xpert MTB/RIF generated results (cycle threshold values) listed. Unless stated otherwise, data is displayed as means and standard deviation (SD).

## Results

### Patients

**Fig. 1:**
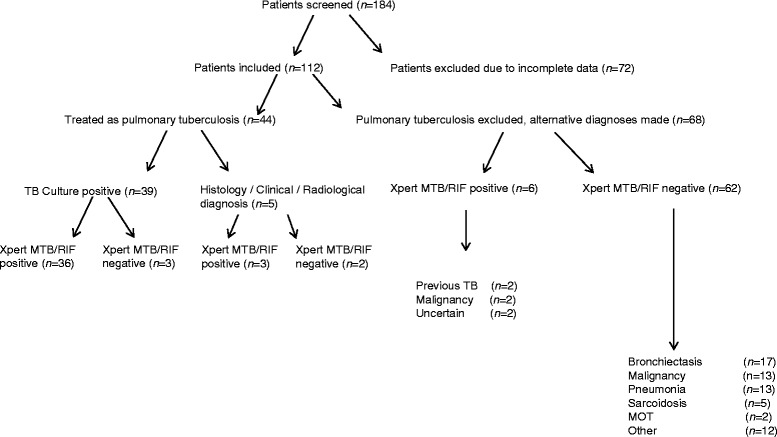
Patients flow through the study. *MOT* Mycobacterium other than tuberculosis

We screened 184 patients with suspected pulmonary tuberculosis during the study period, and included 112 patients (age 44.4 +/− 15.9 years, 54 males) with complete records (Fig. 1). A total of 132 lobes were sampled in these patients.

A total of 44 patients were ultimately treated for pulmonary tuberculosis, based on positive culture (*n* = 39), histological or cytological features (*n* = 2) and on clinical and radiological grounds with clinical response to treatment (*n* = 3). Alternative diagnoses for the remaining 68 patients are summarised in Fig. 1.

Radiographic features observed in the study population included cavitatory disease (*n* = 12), consolidation (*n* = 24), diffuse nodular infiltrates (*n* = 13), alveolar infiltrates (*n* = 35), pulmonary mass lesions (*n* = 13), pleural effusions (*n* = 9) and isolated lymphadenopathy (*n* = 6).

### Microbiology

Xpert MTB/RIF was positive in 36 of the 39 confirmed cases, compared to smear microscopy that was positive in 16. Only one case of isoniazid mono-resistance was identified in the 39 cases who were culture positive, with no cases of Rifampicin resistance. The sensitivities, specificities, PPVs and NPVs for culture, microscopy and Xpert MTB/RIF are summarised in Table 1. Xpert MTB/RIF had a sensitivity of 92.3 % (95 % CI 78–98 %) for PTB, which was superior to that of smear microscopy (41 %; 95 % CI 26–57.8 %, *p* = 0.005). The specificities of Xpert MTB/RIF and smear microscopy were 87.7 % (95 % CI 77.4–93.9 %) and 98.6 % (95 % CI 91.6 %–99.9 %) respectively. The diagnostic efficiency of Xpert MTB/RIF was 89.3 %. Cycle threshold values for the patients diagnosed with PTB varied between high positive (*n* = 8), medium (*n* = 12), low (*n* = 11), and very low positive (*n* = 5).

**Table 1:** Sensitivity, specificity, positive predictive value and negative predictive value of culture, microscopy and Xpert MTB/RIF performed on lavage specimens

	Smear Microscopy		Xpert MTB/RIF		Adjusted Xpert MTB/RIF^a^	
	%	95 % CI	%	95 % CI	%	95 % CI
Sensitivity	41.0	26 – 57.8	92.3	78 – 98	88.6	74.6 – 95.7
Specificity	98.6	91.6 – 99.9	87.7	77.4 – 93.9	91.2	81.1 – 96.4
PPV	94.1	69.2 – 99.7	80.0	64.9 – 89.9	86.7	72.5 – 94.4
NPV	75.8	65.7 – 83.7	95.5	86.6 – 98.8	92.5	82.7 – 97.2

*95 % CI* 95 % confidence interval, *PPV* positive predictive value, *NPV* negative predictive value

^a^Secondary analysis (including cases diagnosed on clinical, histological and radiological means)

Nine Xpert MTB/RIF positive, culture negative cases were identified (Table 2). Of these, the majority had very low positive Xpert MTB/RIF results (*n* = 7) and only three were ultimately treated for suspected pulmonary tuberculosis.

**Table 2:** Xpert MTB/RIF positive, culture negative cases

Case	Previous PTB	Ct value	Treated for PTB	Alternative diagnoses/comments
1	Unknown	Low	Yes	Confirmed pleural tuberculosis
2	No	Very low	Yes	Takayashu’s arteritis
3	Unknown	Very low	Yes	Clinical response to PTB treatment
4	Yes	Medium	No	Surveillance bronchoscopy to exclude bronchial stenosis 1 year after PTB
5	Yes	Very low	No	Repeat bronchoscopy culture and Xpert MTB/RIF negative
6	Unknown	Very low	No	Squamous cell carcinoma of the lung
7	No	Very low	No	Squamous cell carcinoma of the lung
8	No	Very low	No	Symptoms resolved with treatment
9	Yes	Very low	No	Culture positive for *Mycobacterium avium intracellulare*

*PTB* pulmonary tuberculosis, *Ct value* cycle threshold value

## Discussion

In this study, performed in a population with a high pre-test probability for pulmonary tuberculosis, we found Xpert MTB/RIF to have a sensitivity, specificity and negative predictive value of around 90 %, and a positive predictive value of approximately 80 %. Xpert MTB/RIF had a very high diagnostic efficiency of 89.3 %. The majority of Xpert MTB/RIF positive, culture negative results were observed in cases with very low positive Xpert MTB/RIF results and a past history of pulmonary tuberculosis.

The sensitivity observed in the present study is on par with sensitivities recently reported in two studies (ranging from 82–93 %), but the specificity is marginally lower than the reported 96–100 % [7, 8]. The studies reported by Lee *et al.* and Theron *et al.* included similar numbers of patients diagnosed with pulmonary tuberculosis, and utilised comparable diagnostic criteria for the diagnosis of pulmonary tuberculosis [7, 8].

We identified 9 cases of Xpert MTB/RIF positive culture, negative cases, of which three were treated for pulmonary tuberculosis. Current evidence suggests that pulmonary tuberculosis treatment within the preceding 5 years can potentially RIF/RIF [14, 15]. Up to 27 % of patients have been reported to remain sputum Xpert MTB/RIF positive 26 weeks after successful anti-tuberculous treatment was initiated [16, 17]. Due to the nature of the polymerase chain reaction test, Xpert MTB/RIF amplifies any DNA whether it originates from alive or dead bacilli. Therefore it cannot be assumed, solely on the basis of the test, that a positive result equates to active disease [12, 18]. It is also unclear whether the bacterial load in bronchoscopy samples in patients with latent infection or recent exposure would reach this limit and potentially result in a very low positive Xpert MTB/RIF test result. More evidence is needed on the interpretation of Xpert MTB/RIF in the setting of recently treated tuberculosis, which may be the most important cause of false positive results.

No cross-reactivity has been reported between Xpert MTB/RIF and multiple bacteria, viruses, fungi or mycobacteria species other than tuberculosis and should therefore not be the reason for false positive results [19, 20]. We observed a single case of false positive Xpert MTB/RIF in a patient with confirmed *Mycobacterium avium intracellulare* infection. It is likely that dual pathology (including undiagnosed previous tuberculosis) and laboratory contamination may have been present in this case, as well as the other cases not treated as pulmonary tuberculosis, a finding that has been reported in similar settings with a high HIV prevalence [21-23].

It should be noted that 7 of the 9 Xpert MTB/RIF positive culture negative cases had very low positive test results. Some correlation between Xpert MTB/RIF generated quantitative information and bacterial load and disease severity has been described [21, 22]. Xpert MTB/RIF’s calculated analytical limit of detection has been reported as 131 cfu/ml (95% CI 106.2–176.4). The relationship between Xpert MTB/RIF generated cycle threshold values, bacterial load and smear microscopy grades has shown that very low positive test results (cycle threshold values >28 cycles) correlate with negative microscopy and low positive values (22–28) with scanty positive smear microscopy [7, 21, 22]. This also correlated to the lower end of time-to-positivity of cultures. More research is required to aid in the interpretation of Xpert MTB/RIF with very low positive results, which in clinical practice, may be interpreted in the same light as a negative or, at best, scanty positive smear microscopy result. Our observations suggest that very low positive results require confirmation by culture or other means in cases with a low pre-test probability. Cut-off values for positivity should potentially vary between high and low endemic tuberculosis areas with lower cycle threshold values accepted as positive in high endemic areas. Moreover, clinicians practicing in high burden settings should be aware that past tuberculosis and dual pathology should be considered in cases with unexpected positive Xpert MTB/RIF.

The present study has several limitations. The retrospective nature limited the data collection to cases with complete clinical, radiological and microbiological data, and precluded any analyses on the excluded patient population. Some cases with false negative cultures may have gone undetected, and the true sensitivity may be different. We were also not able to document the true impact of Xpert MTB/RIF performed on bronchial lavages on the time to diagnosis or treatment, which is arguably of even greater clinical relevance.

## Conclusion

Xpert MTB/RIF has a higher sensitivity than smear microscopy and similar specificity for the immediate confirmation of pulmonary tuberculosis on specimens obtained from bronchial washing, and should routinely be utilised in patients with a high clinical suspicion of pulmonary tuberculosis. Positive Xpert MTB/RIF, culture negative results should be interpreted in the clinical context, and ideally be confirmed with additional tests and/or follow-up.

## Abbreviations

CI: Confidence Interval
DNA: Deoxyribonucleic acid
HIV: Human Immunodeficiency Virus
NaOH: Sodium Hydroxide
NPV: Negative Predictive Value
MTB/RIF: Mycobacterium tuberculosis/Rifampicin
PPV: Positive Predictive Value
PTB: Pulmonary tuberculosis
WHO: World Health Organisation
